# Effects of pH and Temperature on the Stability of Fumonisins in Maize Products

**DOI:** 10.3390/toxins9030088

**Published:** 2017-03-01

**Authors:** Marcin Bryła, Agnieszka Waśkiewicz, Krystyna Szymczyk, Renata Jędrzejczak

**Affiliations:** 1 Department of Food Analysis, Prof. Waclaw Dabrowski Institute of Agricultural and Food Biotechnology, Rakowiecka 36, 02-532 Warsaw, Poland; krystyna.szymczyk@ibprs.pl (K.S.); renata.jedrzejczak@ibprs.pl (R.J.); 2 Department of Chemistry, Poznan University of Life Sciences, Wojska Polskiego 75, 60-625 Poznan, Poland; agat@up.poznan.pl

**Keywords:** fumonisins, conjugated fumonisins, LC-TOF-HRMS, maize products, stability of fumonisins, pH and temperature conditions

## Abstract

This paper is a study of the stability of fumonisins in dough based on maize flour prepared in a phosphate buffer with a pH of 3.5, 5.5 or 7.5 and baked at a temperature within the range of 100–250 °C. Buffers with various pH values were tested, since it is well-known that pH may significantly influence interactions of fumonisins with other substances. A standard analytical procedure was used to determine the concentration of free fumonisins. Hydrolysis in an alkaline medium was then applied to reveal the hidden forms, while the total fumonisins concentations was determined in another measurement. The total concentration of fumonisins was statistically higher in pH = 3.5 and pH = 5.5 than the concentration of free fumonisins; no similar difference was found at pH = 7.5. The applied phosphate buffer pH 7.5 may enhance solubility of fumonisins, which would increase extraction efficiency of free analytes, thereby decreasing the difference between concentrations of total and free fumonisins. Hydrolysed B_1_ fumonisin (HFB_1_) and partially hydrolysed B_1_ fumonisin (isomers a and b: PHFB_1a_ and PHFB_1b_, respectively) were the main investigated substances. For baking temperatures below 220 °C, fumonisins were slightly more stable for pH = 5.5 than for pH = 3.5 and pH = 7.5. In both of these latter cases, the concentration of partially hydrolysed fumonisins grew initially (up to 200 °C) with an increase in the baking temperature, and then dropped. Similar behaviour was observed for free HFB_1_, which may suggest the following fumonisin degradation mechanism: initially, the tricarballylic acid (TCA) groups are removed from the molecules, and next, the HFB_1_ molecules disintegrate.

## 1. Introduction

Maize (*Zea mays L.*), the third most important crop cultivated on Earth after wheat and rice, is a popular source of nourishment for inhabitants of various regions of the world as well as one of the most popular raw materials used in animal fodder [1]. However, maize is very often infested with *Fusarium verticilloides* and *Fusarium proliferatum* fungi, which produce toxic secondary metabolites—a group of substances collectively called fumonisins. These toxins are very often found in maize-based food products [2,3]. Fumonisin B_1_ (FB_1_), fumonisin B_2_ (FB_2_) and fumonisin B_3_ (FB_3_) are the most frequent ones, among which FB_1_ dominates. Consumption of corn grain contaminated with fumonisins may induce leukoencephalomalacia in horses, pulmonary oedema in swine, liver injury, nephrotoxicity, liver cancer in rats, and arteriosclerosis in primates other than humans. Epidemiologic studies in some South Africa populations have shown a high correlation between the fumonisin level in food and the oesophagus cancer incidence rate [4,5,6]. For this reason, the International Agency for Research on Cancer (IARC) has classified FB_1_ in toxicity class 2B as probably carcinogenic for people [7], while the FAO/WHO JECFA Committee and the European Commission Scientific Committee for Food have set a tolerable daily intake level of FB_1_, FB_2_, FB_3_ or their combination at 2 µg per kg body weight. The EU has defined the maximum permissible levels for the sum of FB_1_+FB_2_ in maize and maize-products as 200–4000 µg·kg^−1^ depending on the foodstuff. Similar US regulations set 2000–4000 µg·kg^−1^ levels for the sum of FB_1_ + FB_2_ + FB_3_ depending on the foodstuff [6].

FB_1_ is a diester of propane-1,2,3-tricarboxylic acid (TCA) and 2-amino-12,16-dimethyl-3,5,10,14,15-pentahydroxy-icosane, in which the C-14 and C-15 hydroxyl groups form esters with the TCA group at the chain end. FB_2_ and FB_3_ are analogues of FB_1_. The amino groups and TCA groups of fumonisins facilitate interactions with other compounds, resulting in various bound forms of fumonisin [8,9,10].

Fumonisins are generally considered to be thermally stable. Nevertheless, their stability may depend on conditions that are more or less favourable for chemical reactions. The paths along which they are transformed in various technological processes applied in food processing were studied by Bullerman et al. [11], Castells et al. [12], De Girolama et al. [13], and Humpf and Voss [14]. Still, transformation mechanisms have not been completely clarified. Some authors have suggested that fumonisins may react with some components of the food matrix during technological processes [14]. Others are of the opinion that hidden fumonisins may be formed already in raw (unprocessed) maize due to supra-molecular interactions with biopolymers [9,10]. In their recently published paper, Lazzaro et al. [15] suggested that non-covalent interactions with food macro-components are based on some physical interactions between mycotoxins and food components. Since hidden fumonisins are not detectable by means of standard analytical procedures [16], but might be released inside the human gastrointestinal tract, they may cause underestimation of the real food safety hazard and therefore are a concern from a toxicological point of view [8]. The presence of hidden fumonisins in raw maize and their ability to form covalent bonds with other compounds during technological processes make the task of understanding the reaction mechanisms significantly more difficult. New reports that raw maize may contain fumonisin derivatives formed by the esterification of fatty acids in reactions most likely assisted by some fungi enzymes as well as some *N*-acyl derivatives formed in similar conditions [17,18] have only compounded that difficulty.

For that reason, in this work, it was decided to determine both the concentration of free fumonisins (using the standard analytical procedure) and the total concentration of all fumonisins, including those released from hidden forms during alkaline hydrolysis reaction. Hydrolysed forms of fumonisins produced during the reaction may be assayed using a liquid chromatograph coupled with a mass spectrometer. To the best of our knowledge, this is the first attempt to study the stability of free and hidden fumonisins in buffers with various pH values applied when preparing dough from maize-based flour and during dough baking at various temperatures.

## 2. Results and Discussion

The concentrations of the FB_1_/FB_2_/FB_3_ sum of free fumonisins and the FB_1_/FB_2_/FB_3_ sum of total fumonisins determined in maize flour and maize dough prepared in three different buffers (pH = 3.5, 5.5, and 7.5) at T = 25 °C and baked at eight temperatures (100, 130, 160, 180, 200, 220, 230, and 250 °C) are shown in Table 1. The penultimate column shows the concentrations of hidden/bound fumonisins, calculated as differences between total and free fumonisins. To facilitate the comparison of results concerning flour and dough, concentrations in the dough were normalized to the amount of flour used to prepare a given dough.

**Figure 1:**
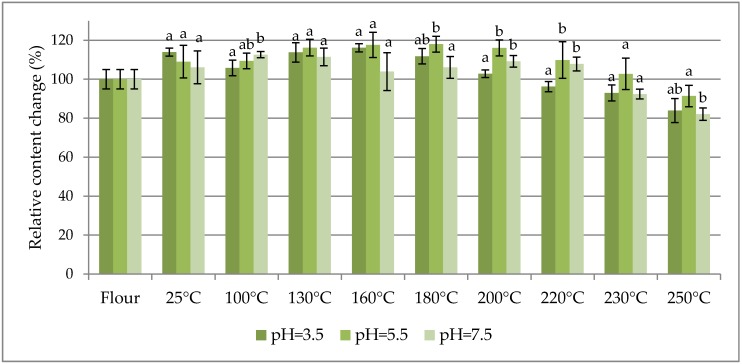
The percentage change in content of the sum of free fumonisins (increase or decrease) normalized to their concentration in flour (as 100%) vs. baking temperature of the dough prepared in three different buffers (pH = 3.5, 5.5, and 7.5). Letters denote statistically homologous groups.

**Figure 2:**
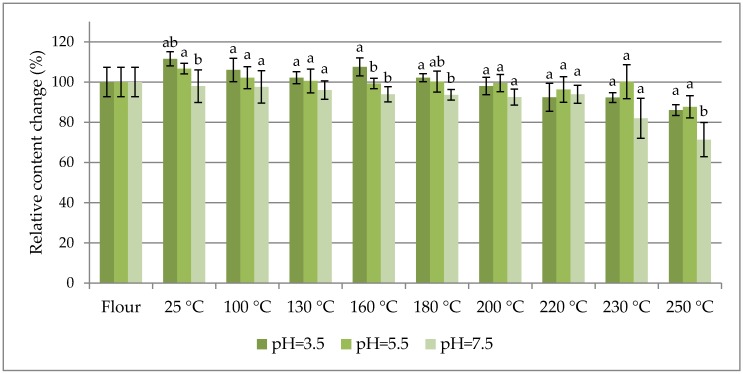
The percentage change in content of sum of total fumonisins normalized to their concentration in flour (as 100%) vs. baking temperature of dough prepared in three different buffers (pH = 3.5, 5.5, 7.5). Letters denote statistically homologous groups.

**Figure 3:**
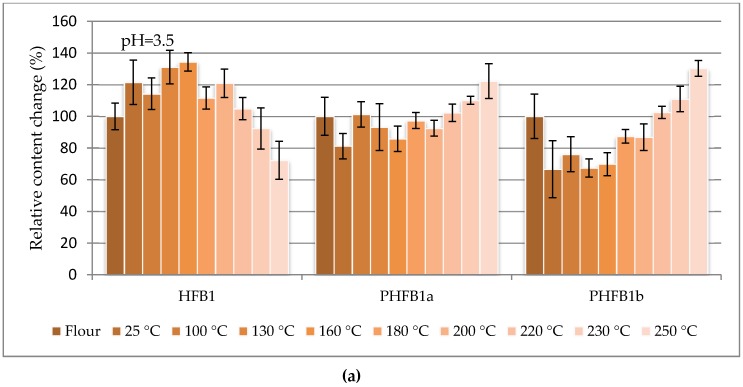

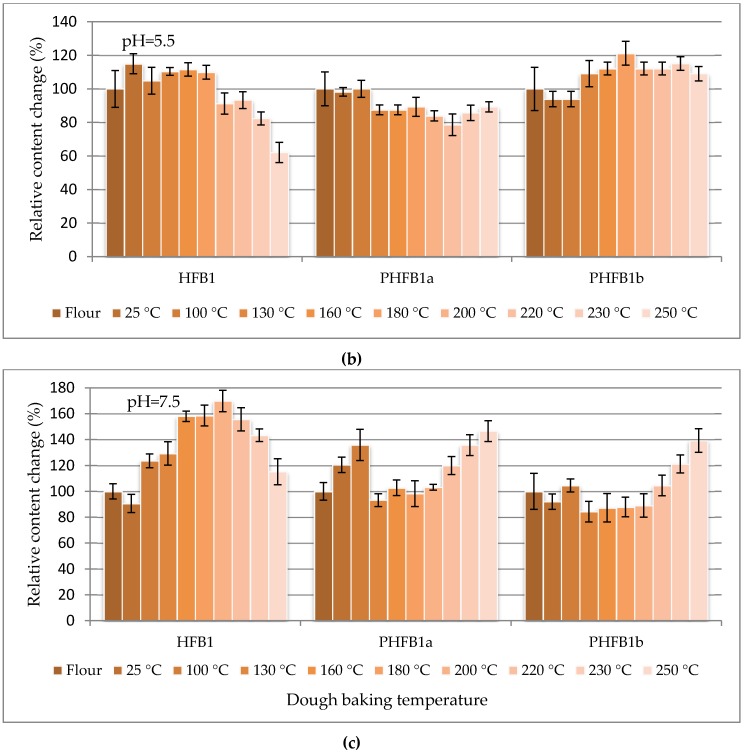
The percentage change in content of HFB_1_, PHFB_1a_ and PHFB_1b_ normalized to their concentration in flour (as 100%) vs. baking temperature of the dough prepared in various buffers (pH = 3.5 (**a**), pH = 5.5 (**b**), and pH = 7.5 (**c**)) charted on the basis of the intensity of the given fumonisin peak normalized to the intensity of the ^13^C-FB_1_ internal standard peak. HFB_1_: hydrolysed B_1_ fumonisin; PHFB_1a_: partially hydrolysed fumonisin B_1_ (isomer a); PHFB_1b_: partially hydrolysed fumonisin B_1_ (isomer b).

The percentage change in content of selected fumonisins normalized to their concentration in flour (as 100%) are charted vs. the dough baking temperature in Figure 1, Figure 2 and Figure 3 to facilitate the study of fumonisin temperature stability. Figure 1 shows the sums of free fumonisins (three curves for the investigated pH values), Figure 2 shows the sums of the total fumonisins (three curves for the investigated pH values), and Figure 3 shows three curves for hydrolysed B_1_ fumonisin (HFB_1_), partially hydrolysed B_1_ fumonisin (isomers a and b: PHFB_1a_ and PHFB_1b_, respectively) charted separately for pH = 3.5 (top), pH = 7.5 (middle) and pH = 5.5 (bottom). The ordinate of the first (flour) data point in each chart is 100% by definition. All other points represent the data for dough starting from room temperature (RT) data charted at 25 °C. The positions of points along the abscissa axis do not represent the baking temperatures.

As seen in Table 1, the maximum contribution of hidden/bound fumonisins to the total fumonisins did not exceed 20% within the entire range of the investigated dough baking temperatures. In other words, at least 80% of the fumonisins in the analysed maize were in the free form. As can be seen in Table 1, the concentration of free fumonisins at 25 °C is higher in buffers containing contaminated flour (4542 ± 287; 4346 ± 363; 4230 ± 356 µg·kg^−1^, for pH 3.5, 5.5 and 7.5 buffers, respectively) as compared with the concentration of free fumonisins determined directly in the flour (3937 ± 205 µg·kg^−1^). Nevertheless, single-variable ANOVA revealed that the total fumonisin concentration was statistically higher than the free fumonisin concentration in dough prepared in pH = 3.5 and pH = 5.5 buffers, while it was not higher in the dough prepared in the pH = 7.5 buffer. Thereby, the hidden-to-free fumonisin concentration ratios turned out to be the lowest for that model (0.03–0.10), while in other cases the averages were within the 0.08–0.25 range. Concentrations of free hydrolysed/partly hydrolysed HFB_1_ compounds were calculated from the area of the peak for a given analyte normalized to the area of the ^13^C-FB_1_ peak. As seen in Figure 1, Figure 2 and Figure 3, the thermal stabilities of all the investigated fumonisins in dough prepared in buffers at all of the investigated pH values (except for several HFB_1_, PHFB_1a_, and PHFB_1b_ points for dough prepared in the pH = 7.5 buffer) were better than ±40% within the entire range of the investigated dough baking temperatures. In dough prepared in pH = 7.5 buffer, the highest percentage change in content of HFB_1_ (reached at a 200 °C baking temperature) was merely 170%. Notwithstanding the above, a slight degradation trend was observed for temperatures higher than approximately 220 °C.

The high thermal stability of free fumonisins has been reported by many authors [14,19,20,21,22]. However, practically no data on the thermal stability of hidden, bound, or conjugated forms are available in the literature. A majority of studies conducted several years ago were focussed on the concentration of free fumonisins during food processing. It is widely accepted that the fumonisin concentration noticeably drops during thermal processing involving temperatures above 150 °C [23,24,25,26]. Bullerman and Bianchini [19] reported degradation of over 90% of the initial amount of fumonisins after heating at 175 °C for 60 min regardless of pH.

Some authors have demonstrated that the thermal stabilities of fumonisins might well depend on the amount of water in the medium containing the fumonisins, as fumonisins seem to be much more susceptible to destruction in dry environments rather than in wet ones [20]. Jackson et al. reported a drop of 16%–28% of the initial FB_1_ concentration of 5 mg/kg after baking corn muffins at 175–200 °C for 20 min [21]. The concentration dropped more at the surfaces of the muffins than within their ground tissue. Castelo et al. [22] reported no reduction of the initial FB_1_ concentration of 5 mg/kg after baking corn muffins at 204 °C for 20 min but observed a drop of 48% after baking the muffins at 232 °C. No decrease was observed in another experiment with corn fried at 140–170 °C for 6 min. A decrease of 67% was recorded when frying tortilla chips at 190 °C for 15 min. Avantaggiato et al. [27] reported a 52%–57% loss of FBs in corn muffins baked at 210 °C for 25 min; each muffin in their experiment was spiked with 15 µg of ^14^C-FB_1_ (synthesized by fungi from 1,2-^14^C-sodium acetate) and the FB_1_ standard. The study was repeated using maize flour spiked with 1.56 µg of ^14^C-FB_1_ per muffin; the average observed thermal loss amounted to 51%.

Thermal stabilities of fumonisin standards in aqueous buffers were studied by Jackson et al. [20,21], who reported that FB_1_ and FB_2_ were least stable at pH = 4 in comparison to those at pH = 10 and pH = 7. However, in our experiment with maize dough, the results were quite different, as fumonisins were more stable at pH = 5.5 than at pH = 3.5 and pH = 7.5.

There are many suggestions in the literature on how to interpret apparently conflicting observations. Some authors have suggested that heated fumonisins may form covalent bonds with other compounds. Shier et al. [28] spiked maize flour samples with radioisotope-labelled FB_1_, with 37% of the entire added radioactivity found in the baked products. Out of the remaining part, 46% of the compound was recovered from the protein fraction extracted with a dodecyl sulphate solution. Seefelder et al. [29] in a model experiment showed that heated FB_1_ molecules in maize, formed covalent bonds with polysaccharides and proteins by means of their propano-1,2,3-tricarboxyl acid side chains.

More recent papers reported difficulties encountered by analysts trying to determine free fumonisins in maize and/or maize-based food products applying standard analytical methods. Dall’Asta et al. [9] attempted to determine causes of those difficulties. To that end, fumonisins in five different maize samples were assayed in five independent labs applying various analytical methods (each validated) and different extraction as well as extract purification procedures. The same calibration standards were used in each of the five labs. Despite all the precautions, the results varied. In that situation, the authors believed that the mechanism responsible for the observed disparities involved some supra-molecular interactions of fumonisins with food macroconstituents (proteins and carbohydrates). Such masking behaviour is a kind of physical entrapment found in grain and unprocessed food not subjected to heat treatment [9,16,30]. Heat treatment may induce some compound chemical reactions; thus, it is impractical to separately consider individual fumonisin-masking mechanisms. It may well be that during maize dough baking fumonisins may be liberated from their hidden forms by interacting with food biopolymers either non-covalently or covalently. Both interactions may depend on the pH of the environment.

The single-variable ANOVA applied to our data revealed that concentrations of total fumonisins and free fumonisins in dough prepared in the pH = 7.5 buffer did not differ statistically (i.e., the concentration of hidden/bound fumonisins was very low). This may result from the weak stability of hidden fumonisins under such conditions and/or from conditions unfavourable for the formation of covalent bonds. Results published by Jaksić et al. [31], Pietri and Bertuzzi [32] and Bertuzzi et al. [33] suggest a reasonable hypothesis as to why the concentration of free fumonisins is higher in pH 7.5 buffer. The phosphate buffer used to prepare dough influenced extraction of free fumonisins from the analysed samples, as indicated by the observed free-to-total fumonisin concentration ratios, which practically in all cases were lower than 0.25, the value found directly in flour. The effect was best visible for samples heated in the pH 7.5 buffer. Pietri and Bertuzzi 2012 [32] compared conventional extraction techniques based on mixtures of water (or a phosphate buffer) with organic solvents (methanol, acetonitrile, a mixture of both) with techniques based on phosphate buffers alone. It was shown that the highest extraction efficiency was obtained with phosphate buffers at pH 7.5 [32]. A relatively higher efficiency of free fumonisin extraction from dough may be observed in pH 7.5 buffers. Even if concentrations found for individual buffer pHs (3.5, 5.5, 7.5) were comparable (for respective baking temperatures), no significant differences between concentrations of free and total fumonisins were observed for the pH 7.5 buffer only. The latter observation seems to confirm suggestions made by other researchers, an opinion shared by the authors of this study, that fumonisins are more efficiently extracted in pH environments of 7.5. Based on the pKa values for TCA (3.49, 4.58 and 5.83) and the pKa value for the amino group (which should exceed 9), one can speculate that fumonisins might be “hermaphrodites” within the pH 6.0–9.0 range due to the transfer of a proton from their carboxylate group towards their amino group. It follows that fumonisin functional groups should be more soluble in water within that pH range [32]. Additionally, in alkaline environments, fumonisins may be hydrolysed to forms without one or both TCA groups. It may well be that the intensity of fumonisin hydrolysis increases with the pH of the environment (see the HFB_1_ relative concentrations in Figure 3 bottom compared to the top and middle). Results obtained in this study show that fumonisins degrade preferentially in pH 7.5 buffers (as compared to other models) due to an increased concentration of hydrolysed/partly hydrolysed fumonisins. As can be seen in Figure 3, only the percentage change in content of free HFB_1_ decreased at temperatures above 200 °C, which may be interpreted as initiation of HFB_1_ degradation reactions above that temperature. A decrease of free HFB_1_ concentration with a simultaneous increase of partly hydrolysed forms may indicate that the fumonisin thermal degradation path is in the first stage based on the degradation of fumonisins to partly hydrolysed forms, then to entirely hydrolysed forms. Degradation of free hydrolysed forms is the final stage.

To produce tortillas, maize is cooked in lime water (the nixtamalization process). Hamner and Tinker [34] showed that both HFBs and PHFBs may be released during that process. Additionally, De Girolamo et al. [1] reported an increase of the HFB/PHFB concentration during the process with a simultaneous drop in the free fumonisin concentration by as much as 80%.

Our results concerning the levels of free fumonisins in maize are generally in line with the literature data, in contrast to the results concerning fumonisin thermal stability. Possible causes for the disparity include the dough preparation method, dough baking time and possible dough additives (simple sugars that may facilitate non-enzymatic Maillard’s reaction consisting of the binding of the fumonisin amino group with sugar carbonyl groups). Products of the reaction, such as *N*-(carboxyl-methyl)-FB_1_ or *N*-(deoxy-d-fructos-1-yl)-FB_1_ were also identified in heated food [35]. Newly-discovered analogues 1-(deoxy-fructos-1-yl)-FB_2_ and 1-(deoxy-fructos-1-yl)-FB_3_ also belong to that group [36]. Their *N*-alkyl bonds are not susceptible to either chemical or enzymatic hydrolysis; therefore, the total concentration of fumonisins may apparently decrease during food processing.

## 3. Conclusions

Stability of fumonisins was evaluated when baking maize flour dough prepared in three phosphate buffers: pH 3.5 (model 1), 5.5 (model 2), and pH 7.5 (model 3). The dough was baked for 25 min at various temperatures within the 25–250 °C range. Generally, fumonisins in maize dough baked at temperatures up to 250 °C were stable. Their concentration did not change by more than ±40% within the entire temperature range, except for a few HFB_1_/PHFB_1a_/PHFB_1b_ points for dough prepared in the pH = 7.5 buffer. A slight degradation trend was observed above 220 °C. The largest change to 170% was noted for HFB_1_ in dough prepared in the pH = 7.5 buffer, and that maximum was reached at 200 °C, then HFB1 concentration decreased. That may suggest the following fumonisin degradation mechanism: first TCA groups are removed from the molecule, then the HFB_1_ molecule disintegrates.

Among the three investigated buffer pH values in which the dough was prepared (3.5, 5.5, and 7.5), the thermal stability was highest at pH = 5.5. At that latter pH, within the entire range of the investigated baking temperatures, the concentration of the free forms stayed within the +18/−9% range, the total concentration stayed within the +8/−11% range, and the HFB_1_ concentration stayed within the +1/−23% range.

The maximum contribution of hidden/bound fumonisins to the total fumonisins did not exceed 20% within the entire range of the investigated dough baking temperatures. In other words, at least 80% of the fumonisins in the analysed maize were found in the free form. Nevertheless, single-variable ANOVA revealed that the total fumonisin concentration was statistically higher than the free fumonisin concentration in dough prepared in pH = 3.5 and pH = 5.5 buffers, while it was not in dough prepared in the pH = 7.5 buffer. The reason is a low average value of the hidden and bound to free fumonisin ratios in those samples (0.03–0.10), when compared to a larger ratio for other models (0.08–0.25). Probably, it reflects a larger solubility of fumonisins in the pH 7.5 buffer, and consequently a larger extraction of the analytes from the samples. That is in agreement with data available in the literature. 

It seems that thermal processing up to 250 °C practically does not degrade fumonisins in maize. Rather, the structure of fumonisin molecules may change into partially hydrolysed forms, which may influence living organisms similarly to free forms.

## 4. Experimental Section

### 4.1. Reagents and Other Chemicals

High-purity mycotoxin standards supplied by Romer Labs (Tulln, Austria) included the following: 50 µg·mL^−1^ FB_1_; 50 µg·mL^−1^ FB_2_; 50 µg·mL^−1^ FB_3_; 25 µg·mL^−1 13^C-labelled FB_1_ (^13^C-FB_1_); 10 µg·mL^−1 13^C-FB_2_; and 10 µg·mL^−1 13^C-FB_3_. All supplied standards were dissolved in a 1:1 acetonitrile:water mixture. 

HPLC-grade methanol, acetonitrile and dichloromethane were purchased from Rathburn Chemicals Ltd. (Walkerburn, UK). Analytical-grade acetic acid, formic acid and potassium hydroxide were obtained from POCh (Gliwice, Poland). The Chromasolv LC-MS-grade water was purchased from Fluka (Buchs, Switzerland). Samples were filtered through 0.2-µm mesh, 4-mm-diameter nylon syringe filters purchased from Phenomenex (Torrance, CA, USA).

### 4.2. Buffers

Phosphate buffers with pH values of 3.5, 5.5, and 7.5 were used. The two former buffers were prepared based on a 0.1 M solution of disodium phosphate, while the latter was prepared based on a 0.1 M solution of sodium phosphate. Ortho-phosphoric acid (2%) was added until the required pH was obtained (in the presence of an electrode in the two former cases). The pH was controlled with the help of a CPC-505 pH meter manufactured by Elmetron (Zabrze, Poland).

### 4.3. LC-MS Instrument

An Acquity H-Class high-performance liquid chromatograph coupled to an LCQ Premiere XE high resolution time-of-flight mass spectrometer (Waters, Milford, MA, USA) was used as the analytical instrument. Analytes were separated on a UPLC C18 Cortecs chromatographic column (2.1 × 100 mm, 1.6 µm; Waters, Milford, MA, USA) with a pre-column at its front. The mobile phase was composed of methanol and water. Both phases contained 0.2% formic acid, while phase B additionally contained 2 mM ammonium formate. The flow rate was 0.4 mL/min. The following gradient was used: 1%–50% A from 0 to 8 min; 50%–95% A from 8 to 11 min; constant 95% A from 11 to 22 min; 95%–1% A from 22 to 22.6 min; and constant 1% A from 22.6 to 27 min. Samples of 2.5 μL were injected onto the column. The mass spectrometer was operated in the positive electrospray ionization mode (ESI). The ion source and desolvation temperatures were 150 °C and 350 °C, respectively. The nebulizing gas (nitrogen) flow rate was 750 L/min and the cone gas flow rate was 20 L/min. The capillary voltage was 3000 V. The V mode of the ion optics was used. The mass spectrometer was calibrated using a standard solution of leu-enkefalin. The following precursor (M + H)^+^ ions (*m*/*z*) were registered for individual analytes: FB_1_, 722.4; ^13^C-FB_1_, 756.5; FB_2_ and FB_3_, 706.4; ^13^C-FB_2_ and ^13^C-FB_3_, 740.5; HFB_1_, 406.4; PHFB_1a and b_, 564.4; PHFB_2a and b_ and PHFB_3a and b_, 548.4; ^13^C-HFB_1_, 428.5; HFB_2_ and HFB_3_, 390.4; and ^13^C-HFB_2_ and ^13^C-HFB_3_, 512.5.

### 4.4. Other Used Apparatuses

In the experiments, the following apparatuses were used: a Hydrolab two-stage reverse osmosis water purification system with ion exchange resin (Wiślina, Poland); an IKA-RV-10 rotary vacuum evaporator (Staufen, Germany); a WŻ-1S impact mill (ZBPP, Bydgoszcz, Poland); a Diax 900 homogenizer (Heidolph, Schwabach, Germany); an MLU-202 grist mill (Bühler GmbH, Uzwil, Switzerland); and an MR-2L grain/flour mixer (Chopin Technologies, Villeneuve-la-Garenne, France).

### 4.5. The Studied Material

Maize naturally polluted with fumonisins was studied. The grain supplied by the Silesian Grain Company was milled in a grist mill. The milled cereal was screened on 1000-µm mesh screens. Five hundred grams of flour and 350 mL of a respective buffer were mixed into dough. The kneaded dough was formed into small pieces using Petri dishes (60 mm diameter, 12 mm high). Separate samples were prepared for each studied pH value (3.5, 5.5, and 7.5) and at each baking temperature (100, 130, 160, 180, 200, 230 and 250 °C). The dough was baked in a convection/drill oven for 25 min after the required temperature was reached. The loss of mass was recorded after baking. Next, the batch was disintegrated using an impact mill. The obtained powder was stored in a freezer at a temperature below –20 °C. The material for the control samples was baked under identical conditions but it was made from flour not polluted with fumonisins.

### 4.6. Sample Preparation

The detailed sample preparation procedure was previously described by Bryła et al. [37].

#### 4.6.1. Free FBs

**Figure 4:**
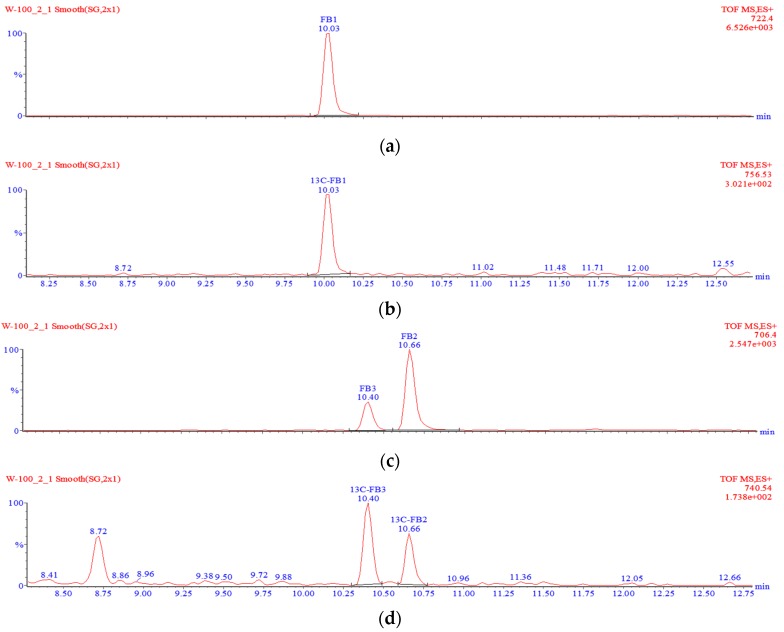
Typical chromatogram of free fumonisins in maize using a time-of-flight (TOF) mass spectrometer: FB_1_ (**a**); ^13^C-FB_1_ (**b**); FB_2_ and FB_3_ (**c**); ^13^C-FB_2_ and ^13^C-FB_3_ (**d**).

A 1.25-g well-ground sample was transferred into a 50-mL beaker, spiked with 10 µL of the ^13^C-FB_1_/^13^C-FB_2_/^13^C-FB_3_ internal standard solution, and homogenized with 10 mL of methanol:acetonitrile:water solution (25:25:50 *V*/*V*/*V*) for 3 min to extract the analytes. The extract was transferred into a 15 mL PP centrifuge tube and centrifuged at 10,730× *g*. Two millilitres of the supernatant were transferred into a round bottom flask and evaporated to dryness using a rotary evaporator. The dry residue was re-dissolved in 1 mL of a 30:70 methanol:water mixture and sonicated in an ultrasound bath. The solution was filtered through a 0.2-µm nylon syringe filter and transferred into some glass vials, followed by analysis with the liquid chromatography–time-of-flight mass spectrometry–high resolution mass spectrometry (LC-TOF-MS) system. A typical chromatogram showing the separation of free fumonisins is shown in Figure 4.

#### 4.6.2. Hydrolysed FBs

**Figure 5:**
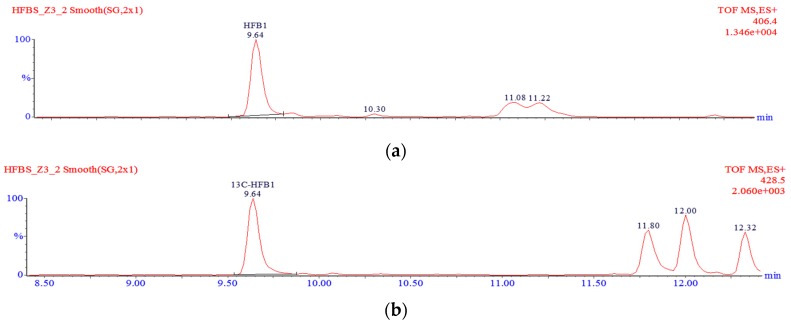

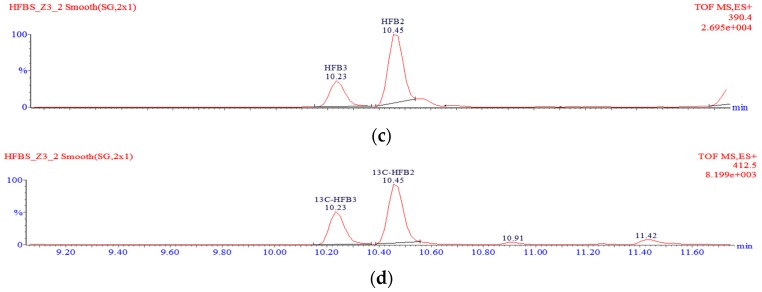
Typical chromatogram of hydrolysed fumonisins in maize using a TOF mass spectrometer: HFB_1_ (**a**); ^13^C-HFB_1_ (**b**); HFB_2_ and HFB_3_ (**c**); ^13^C-HFB_2_ and ^13^C-HFB_3_ (**d**).

A well-ground sample (0.5 g) was transferred into a PP centrifuge tube and spiked with 10 µL of the ^13^C-FB_1_/^13^C-FB_2_/^13^C-FB_3_ internal standard solution. Ten millilitres of a 2 M KOH solution were added, and the tube contents were hydrolysed at room temperature for 24 h. The solution was shaken with 12.5 mL of dichloromethane and centrifuged at 10,730× *g*. Five millilitres of the organic extract were transferred into a round bottom flask and evaporated to dryness using a rotary evaporator operated at 40 °C. Dry residues were dissolved in 1 mL of a methanol-water mixture (30:70 *v*/*v*), filtered through a 0.2-µm nylon syringe filter, transferred into some glass vials and analysed using the LC-TOF-MS system. A typical chromatogram showing the separation of hydrolysed fumonisins is shown in Figure 5.

**Figure 6:**
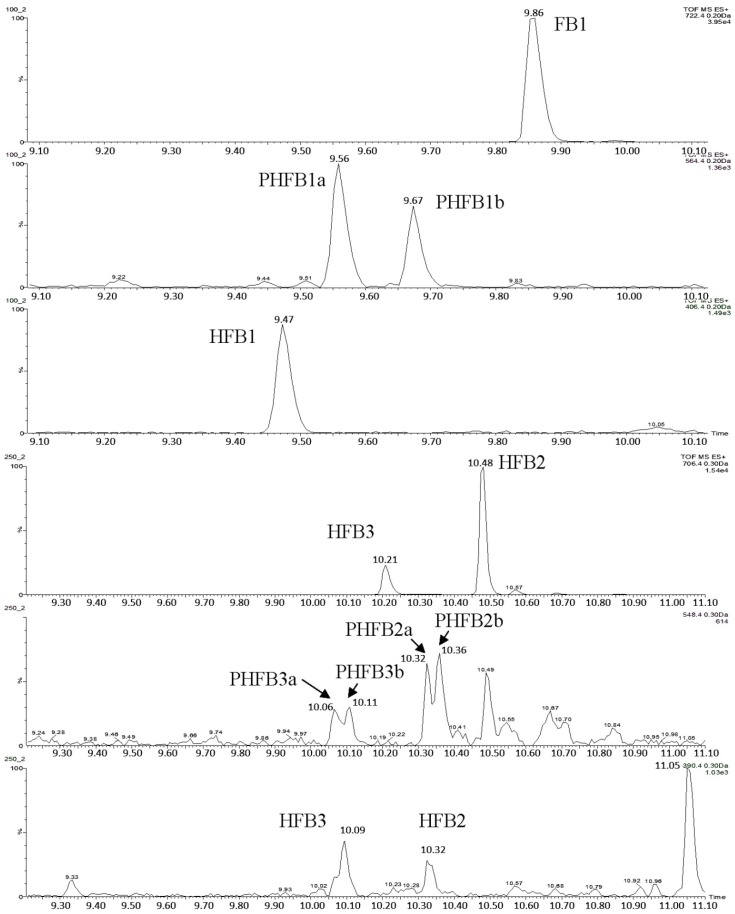
Double peaks produced by partially hydrolysed fumonisins PHFB_1_, PHFB_2_, and PHFB_3_.

If one of the two TCA groups in hydrolysed fumonisin is missing, the molecule is referred to as “partially hydrolysed fumonisin”. Depending on the location of the remaining TCA group, the molecules in question may produce two separate peaks in chromatograms (Figure 6). Such partially hydrolysed fumonisins are hereafter referred to as PHFB_1a_ or PHFB_1b_, PHFB_2a_ or PHFB_2b_, and PHFB_3a_ or PHFB_3b_, respectively.

### 4.7. Calculations and Presentation of the Results

All concentrations were determined against calibration curves taken by measurements of internal ^13^C-labelled standards. The determined HFB_1_, HFB_2_ and HFB_3_ concentrations were re-calculated into the concentration of free fumonisins taking into account the following HFB/FB molecular mass ratios: 0.56 for FB_1_ and 0.55 for FB_2_ as well as FB_3_. The concentration of hidden/bound FBs was determined as the difference between the total FBs and free FBs.

### 4.8. Data Analysis

Statistical data were evaluated using the Statgraphics 4.1 Plus software package (StatPoint Technologies, Inc., Warrenton, VA, USA). One-way ANOVA (analysis of variance) was used to assess the significance of the differences between the determined fumonisin concentrations at the statistical significance level of α = 0.05. All results belonging to a statistically homologous group are marked in figures with the same letter.

### 4.9. Calibration Standards

Standard solutions of FB_1_/FB_2_/FB_3_ fumonisins were dissolved in an extract from a non-polluted sample (to take care of any matrix effects that otherwise might bias the results of the analyses of polluted samples). Calibration curves were made from measurements of a mixture of FB_1_/FB_2_/FB_3_ standard solutions each at a concentration of 5 µg·mL^−1^. Different volumes (2.5, 5, 10, 40, 80, 160, and 240 µL) of the mixture were transferred into 25-mL round bottom flasks containing extract without the solvent, which was removed earlier in a vacuum evaporator. ^13^C-labelled internal standards were also added to the flask in volumes corresponding to the volumes used in analyses of unknown samples, i.e., 2 µL each. The solvent was removed from the internal standard solutions in a vacuum evaporator, and the dry mass was dissolved in 1 mL of a methanol–water (30:70) mixture. The final concentrations of the analytes were 50, 100, 200, 400, 800, 1600, and 2400 µg·kg^−1^, respectively.

Standard solutions of HFB_1_, HFB_2_, and HFB_3_ hydrolysed fumonisins were prepared from 5 µg·mL^−1^ fumonisin standards. Eight 0.5-g portions of a non-polluted sample were transferred into eight glass test tubes and mixed with 10 µL of each of the internal standard solutions (of the same concentration as that used in the analyses of unknown samples). Then, 4, 25, 75, 150, 300, 450, 600, and 900 µL samples of the FB_1_/FB_2_/FB_3_ standard solution were added to individual tubes. Ten millilitres of a 2 M KOH solution was added to each tube, and the tube contents were hydrolysed at RT for 24 h. Subsequent steps were identical as in the procedure to prepare unknown samples. Samples fortified in order to determine the method recovery rate, unknown samples, and calibration standards were prepared simultaneously to keep the hydrolysis conditions (temperature and time) as identical as possible. The final concentrations of analytes (hydrolysis taken into account) were 22, 140, 420, 840, 1680, 2520, 3360, and 5040 µg·kg^−1^, respectively, for HFB_1_ and 22, 138, 413, 825, 1650, 2475, 3300, and 4950 µg·kg^−1^, respectively, for HFB_2_ and/or HFB_3_.

### 4.10. Validation Experiments

Performance of the applied analytical method was evaluated in validation experiments, in which samples of ground maize grain originally free of fumonisins (i.e., samples in which the concentration of fumonisins was below the limit of detection) were fortified with known amounts of individual analytes and then analysed to determine the limit of quantification (LOQ), linear range, recovery (R), and repeatability/precision (characterized by the relative standard deviation, RSD). The following three fortification levels were used: 200, 400, and 600 µg·kg^−1^ for FB_1_, FB_2_, and FB_3_; 420, 840, and 1680 µg·kg^−1^ for HFB_1_; and 413, 765, and 1650 µg·kg^−1^ for HFB_2_ and HFB_3_. Each sample was analysed in triplicate. Characteristic parameters of the applied analytical method are shown in Table 2.

## Figures and Tables

**Table 1:** Concentrations (µg·kg^−1^) of the sum of free fumonisins and sum of total fumonisins determined in maize flour and maize dough prepared in three different buffers (pH = 3.5, 5.5, and 7.5) at T = 25 °C and baked at eight temperatures between 100 and 250 °C. The last column shows the concentrations of hidden/bound fumonisins calculated as the differences between the total and free fumonisins. Concentrations in dough have been normalized to the amount of flour used to prepare the given dough. Letters denote statistically homologous groups. FB: fumonisin B.

Type of Product	Temperature (°C)	Sum of Free FBs (*n* ≥ 3)	Sum of Total FBs (*n* ≥ 3)	Hidden/Bound FBs = Total − Free	Hidden/Bound FBs/Sum of Free FBs
		Concentrations (µg·kg^−1^)			
Flour	-	3937 ± 205 a	4844 ± 354 b	907	0.23
Dough prepared in pH = 3.5 buffer	25	4542 ± 287 a	5402 ± 551 b	860	0.19
	100	4218 ± 471 a	5136 ± 299 b	918	0.22
	130	4536 ± 226 a	4947 ± 147 b	411	0.09
	160	4631 ± 226 a	5210 ± 233 b	579	0.13
	180	4458 ± 175 a	4458 ± 980 b	490	0.11
	200	4399 ± 850 a	4747 ± 201 b	348	0.08
	220	3937 ± 103 a	4477 ± 311 b	540	0.14
	230	3705 ± 151 a	4469 ± 110 b	763	0.21
	250	3346 ± 206 a	4166 ± 113 b	820	0.25
Dough prepared in pH = 5.5 buffer	25	4346 ± 363 a	5167 ± 450 b	821	0.19
	100	4361 ± 174 a	5278 ± 287 b	917	0.21
	130	4634 ± 196 a	5307 ± 313 b	673	0.15
	160	4690 ± 304 a	5267 ± 137 b	577	0.12
	180	4704 ± 192 a	5278 ± 277 b	574	0.12
	200	4627 ± 191 a	5252 ± 225 b	625	0.14
	220	4379 ± 411 a	5056 ± 321 b	677	0.15
	230	4096 ± 330 a	5065 ± 612 b	969	0.24
	250	3643 ± 201 a	4439 ± 245 b	796	0.22
Dough prepared in pH = 7.5 buffer	25	4230 ± 356 a	4348 ± 550 a	118	0.03
	100	4489 ± 720 a	4727 ± 383 a	237	0.05
	130	4444 ± 203 a	4648 ± 213 a	204	0.05
	160	4143 ± 402 a	4550 ± 171 a	407	0.10
	180	4229 ± 236 a	4422 ± 230 a	193	0.05
	200	4352 ± 218 a	4482 ± 178 a	130	0.03
	220	4298 ± 152 a	4550 ± 204 a	251	0.06
	230	3683 ± 920 a	3970 ± 395 a	288	0.08
	250	3270 ± 104 a	3455 ± 404 a	185	0.06

**Table 2:** Characteristic parameters of the applied analytical method.

Compound	LOD	LOQ	Linear Range	Correlation Coefficient	Fortification Level	Sample Count	Recovery R	RSD	Mean Recovery	Mean RSD
	(µg·kg^−1^)	(µg·kg^−1^)	(µg·kg^−1^)	r	(µg·kg^−1^)	n	(%)	(%)	(%)	(%)
FB_1_	4	12.5	12.5–1200	0.9910	200	4	81	11	89	9
					400	4	100	9		
					600	4	87	8		
FB_2_	4	12.5	12.5–1200	0.9872	200	4	82	10	88	8
					400	4	96	5		
					600	4	85	9		
FB_3_	4	12.5	12.5–1200	0.9964	200	4	94	11	95	9
					400	4	90	7		
					600	4	101	10		
HFB_1_	7	22	22–5040	0.9844	420	4	110	9	94	9
					840	4	84	10		
					1680	4	88	8		
HFB_2_	7	22	22–4950	0.9941	413	4	101	11	101	7
					765	4	98	5		
					1650	4	105	5		
HFB_3_	7	22	22–4950	0.9887	413	4	114	6	102	5
					765	4	88	6		
					1650	4	104	5		

Limit of detection (LOD)—concentration at which signal:noise ratio (S/N) is equal to 3; Limit of quantification (LOQ)—concentration at which signal:noise ratio (S/N) is equal to 10. RSD: relative standard deviation; R: recovery.

## Abbreviations

The following abbreviations are used in this manuscript:

FBs	fumonisins
FB_1_	fumonisin B_1_
FB_2_	fumonisin B_2_
FB_3_	fumonisin B_3_
HFB_1_	hydrolysed fumonisin B_1_
HFB_2_	hydrolysed fumonisin B_2_
HFB_3_	hydrolysed fumonisin B_3_
PHFB_1a_	partially hydrolysed fumonisin B_1_ (isomer a)
PHFB_1b_	partially hydrolysed fumonisin B_1_ (isomer b)
PHFB_2a_	partially hydrolysed fumonisin B_2_ (isomer a)
PHFB_2b_	partially hydrolysed fumonisin B_2_ (isomer b)
PHFB_3a_	partially hydrolysed fumonisin B_3_ (isomer a)
PHFB_3b_	partially hydrolysed fumonisin B_3_ (isomer b)
TCA	tricarballylic acid
LC	liquid chromatography
TOF	time-of-flight mass spectrometry
IARC	International Agency for Research on Cancer
HRMS	high resolution mass spectrometry
R	recovery
RSD	relative standard deviation
LOD	limit of detection
LOQ	limit of quantification
